# Monodisperse Polymer Melts Crystallize via Structurally Polydisperse Nanoscale Clusters: Insights from Polyethylene

**DOI:** 10.3390/polym12020447

**Published:** 2020-02-14

**Authors:** Kyle Wm. Hall, Timothy W. Sirk, Simona Percec, Michael L. Klein, Wataru Shinoda

**Affiliations:** 1 Department of Chemistry, Temple University, Philadelphia, PA 19122, USA; simona.percec@temple.edu (S.P.); mlklein@temple.edu (M.L.K.); 2 Institute for Computational Molecular Science, Temple University, Philadelphia, PA 19122, USA; 3 U.S. Army Research Laboratory, Aberdeen Proving Ground, MD 21005, USA; timothy.w.sirk.civ@mail.mil; 4 Department of Materials Chemistry, Nagoya University, Furo-cho, Chikusa-ku, Nagoya 464-8603, Japan; w.shinoda@chembio.nagoya-u.ac.jp

**Keywords:** crystallization, nucleation, stem, dispersity, polyethylene, simulation, molecular dynamics

## Abstract

This study demonstrates that monodisperse entangled polymer melts crystallize via the formation of nanoscale nascent polymer crystals (i.e., nuclei) that exhibit substantial variability in terms of their constituent crystalline polymer chain segments (stems). More specifically, large-scale coarse-grain molecular simulations are used to quantify the evolution of stem length distributions and their properties during the formation of polymer nuclei in supercooled prototypical polyethylene melts. Stems can adopt a range of lengths within an individual nucleus (e.g., ∼1–10 nm) while two nuclei of comparable size can have markedly different stem distributions. As such, the attainment of chemically monodisperse polymer specimens is not sufficient to achieve physical uniformity and consistency. Furthermore, stem length distributions and their evolution indicate that polymer crystal nucleation (i.e., the initial emergence of a nascent crystal) is phenomenologically distinct from crystal growth. These results highlight that the tailoring of polymeric materials requires strategies for controlling polymer crystal nucleation and growth at the nanoscale.

## 1. Introduction

**Figure 1:**
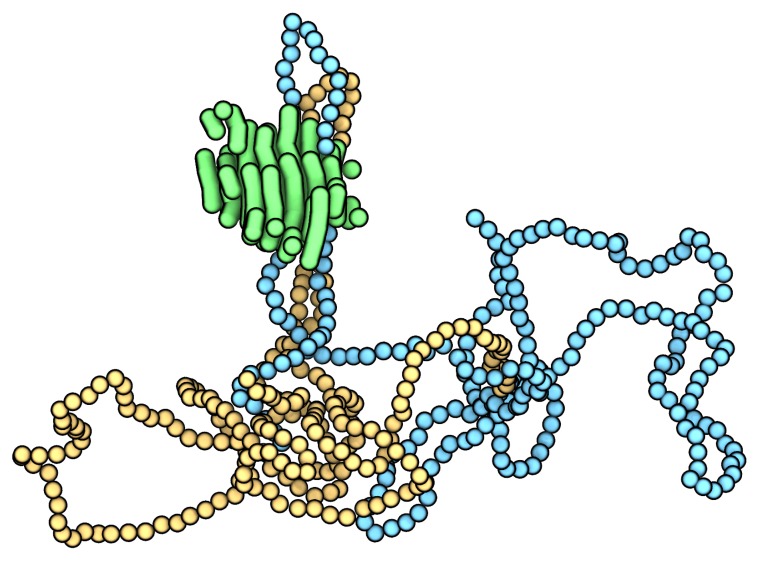
A nascent polyethylene lamella approximately 3 nm across as extracted from one of the nucleation simulations used in this study. The green tubes correspond to the stems composing the crystalline cluster. Note that the cluster is composed of sections of multiple chains. The surrounding polymer melt is not shown for visual clarity. The yellow and blue spheres correspond to two folded polymer chains that traverse the nascent lamella. The folds correspond to the two loops at the top of the cluster. All of the blue and yellow spheres correspond to non-crystalline polymer chain segments.

The properties of polymeric materials—both macroscopic specimens and nanomaterials—are intimately connected to their crystallinity (e.g., see ref. [1]). In particular, many synthetic polymers, such as polyethylene, are semicrystalline. These polymers crystallize via the formation of heterogeneous hierarchical structures composed of nanoscale semicrystalline lamellae, with the exact nature of these structures depending on processing conditions. At the molecular level, the crystallization of such polymer species involves individual polymer chains adopting folded conformations in which crystalline nano-segments of chains, called stems, are connected by disordered folded regions, as shown in Figure 1. The stems constitute the interior of each lamella while the folded regions form their amorphous exterior. This study quantitatively probes stem length distributions, their properties, and their evolution during the initial stages of crystallization (i.e., nucleation) where a nascent nanoscopic crystal (i.e., nucleus) emerges from a supercooled entangled polymer melt.

While there have been many experimental studies on polymer crystallization and nucleation as highlighted by recent reviews [2,3,4], it remains experimentally challenging to simultaneously achieve the nanoscale temporal and spatial resolutions necessary to track the molecular-level evolution of nuclei, rendering many details of nuclei inaccessible (e.g., stem length distributions). In this vein, lamellae are often drawn and discussed as though their constituent stems are of uniform length. Conversely, polymer crystallization models based on statistical mechanics have generally concluded that there are variations in stem lengths, though the extent of such variations is a matter of some debate. For example, in their pioneering theoretical work on polymer crystal nucleation and growth from dilute solutions, Lauritzen and Hoffman [5] emphasized that nascent lamellae exhibit narrow stem length distributions during isothermal crystallization, though their mathematics support the existence of variations in stem lengths. Subsequent work neglected stem length variations (e.g., [6]) while Price [7] came to the conclusion that polyethylene crystals are smooth with only minor 0.1–0.2 nm variations in stem lengths. In contrast, Frank and Tosi [8] concluded that there must be substantial fluctuations in stem lengths during lamellar crystal growth, and that deviations from average lengths increase substantially with greater supercooling (decreasing temperature). Similarly, later work [9] predicted that there can be considerable variations in stem lengths, while also highlighting the connections between broad stem length distributions and lower interfacial free energies. Point [10,11] demonstrated that modeling the initiation of a new layer of stems on a crystalline lamellar substrate as a sequential process with intermediates involving variable stem lengths enables recovery of the experimentally observed relationship between lamellar thickness and degree of supercooling. Importantly, Point’s treatment with variable stem lengths avoided the nonphysical divergence of stem lengths at large undercooling (i.e., the so-called δl catastrophe) that had plagued previous work [6]. Sadler and colleagues [12,13] connected polymer crystal growth to variations in stem lengths, with a particular emphasis on how stem length variations give rise to tapered growth faces and an entropic barrier to polymer crystal growth. While disparate models indicate that stem length variations are possible and potentially significant, these models do not consider the entangled nature of molten polymer chains, nor polymer chain dispersity, and generally neglect the topological effects of folds. Moreover, most models focus on crystal growth rather than the initial formation of a crystal nucleus—so-called primary nucleation.

With molecular simulations, one can directly study the molecular-level nanoscopic details of polymer crystal nuclei and stems during polymer crystallization. Previous in silico studies have probed stem-related properties and phenomena during crystallization, including the lamellar thickness of nuclei [14,15], intra-lamellar stem reordering mechanisms [16], stem growth rates as a function of stem length [16,17], the structural details of crystal growth fronts [16,18,19], and connections between average stem lengths and crystallization conditions (e.g., temperature and system composition) [20,21,22,23]. In particular, Doye and Frenkel [24] studied the distribution of stem lengths during the formation of crystalline layers from solution on polymer crystal growth faces of varying thickness, and thereby revealed that stem lengths can vary substantially. Doye and Frenkel [25] subsequently reexamined Sadler and Gilmer’s two-dimensional model of polymer crystallization [12,13], and came to similar conclusions. More specifically, the stem length probability distribution for a bulk crystal is approximately symmetric about the average thickness of the mature crystal (the maximum of the distribution) [25]. This work by Doye and Frenkel also highlighted that the outer layers (i.e., the surfaces of growing crystals) exhibit stem length distributions that are shifted towards short stems. A subsequent characterization [16] of stem length distributions and their evolution in the context of polyethylene crystal growth reinforced and further elucidated that the central mature regions of lamellae can exhibit substantial variations in stem lengths. Still others have extracted system-wide stem length distributions following crystallization (e.g., [21]). Based on the visual inspection of snapshots from nucleation simulations, Meyer and Müller-Plathe [26] noted in passing that nuclei could be composed of stems of varying length and contain a variable number of stems. Similarly, images of polymer crystal nuclei from previous work [15,17,19,20,23,27,28,29,30,31,32,33,34,35,36] exhibit stem length variability, and sometimes quite pronounced variability, though these studies did not directly explore nor quantify the stem length distributions of nuclei. More recently, Hagita et al. [37] demonstrated that stem length probability distributions broaden as linear and ring polyethylene melts crystallize, though multiple nuclei of differing size formed in their systems, obfuscating the structural details of individual nuclei. Importantly, while both theoretical and computational studies indicate that polymer crystallization does involve variable stem lengths, it remains an outstanding challenge to quantitatively analyze: (1) stem length distributions of individual polymer crystal nuclei; (2) the properties of these distributions; and (3) their evolution during crystal nucleation. We address this challenge herein on the basis of large-scale coarse-grain molecular simulations of polymer crystal nucleation in supercooled prototypical polymer melts, specifically polyethylene melts.

## 2. Methods

In order to quantitatively probe the characteristics of stem length distributions during polymer crystal nucleation, we performed microsecond molecular dynamics simulations of crystal nucleation in monodisperse polyethylene melts composed of *n*-C_720_H_1442_ chains. The methodological details of the simulations are the same as our previous work [38], so only a summary of the calculations are provided herein (see [38] for more details). All simulations were conducted using the Large-scale Atomic/Molecular Massively Parallel Simulator (LAMMPS) [39]. The *n*-C_720_H_1442_ chains were represented using the CM and CT coarse-grain beads from the coarse-grain Shinoda-DeVane-Klein (SDK) molecular model [40]. For reference, the CM and CT beads were used to represent –(CH_2_)_3_– and –CH_2_CH_2_CH_3_ units along the polyethylene backbones. By comparing SDK-based results for molten polyethylene and polyethylene crystallization to results from experiments and atomistic simulations, we have previously demonstrated that the SDK model is an appropriate model for studying polyethylene systems [41]. Due to their coarse-grain nature, molten SDK polyethylene chains diffuse ∼4.07 times faster than those in experimental samples, as discussed in our previous work [41,42]. As such, all simulation times reported in this study correspond to scaled times (i.e., internal LAMMPS times × 4.07), unless otherwise stated, in order to facilitate comparisons with experimental timescales.

A *n*-C_720_H_1442_ melt consisting of 400 chains under periodic boundary conditions was prepared and equilibrated at 500 K. Ten independent configurations were selected from the high-temperature simulation and used to generate ten independent metastable melts at 300 K. These melts were in turn quenched to 285 K, and each melt was simulated at 285 K for ∼4 μs under isobaric-isothermal conditions (i.e., NPT conditions). At 285 K, homogeneous nucleation occurred on the microsecond timescale, though not all of the melts crystallized during their ∼4-μs simulation window. The methodological details of the nucleation simulations are provided in Table 1. For each simulation, the equations of motion of Shinoda et al. [43] were used to temporally evolve particle positions in accordance with the details provided in Table 1. System configurations were saved every 20,000 time steps during each nucleation simulation in order to analyze the crystallization process.

Consistent with previous work [14,18,26,34,38,41,42,47,48,49,50,51], polymer crystallization was monitored using the $P_{2}$ order parameter. Note that the $P_{2}$ order parameter is sometimes alternatively referred to as *S* or $P_{l}$ depending on how it is calculated and on author preferences. In any case, the order parameter provides a quantitative assessment of the alignment between polymer chain segments within a system. The $P_{2}$ analysis was performed according to the protocol detailed in [38,42]. In brief, coarse-grain beads with $P_{2}\geq 0.85$ were labelled crystalline, and cluster analysis was performed on crystalline coarse-grain beads to extract nuclei. This analysis yielded over four million nanoscale nuclei. The stems composing each nucleus were extracted by searching for sets of contiguous crystalline coarse-grain beads along the backbone of each constituent polymer chain. As such, a stem could be as short as a single bead, which corresponds to three methylene units.

It is reasonable to include such single-bead stems based on theoretical considerations and in silico results. First, theoretical treatments [7,8,9,10,11,12,13] of polymer crystallization generally support variations in stem length with a number of studies—including the work of Sadler and Gilmer [12,13] and that of Point [10,11]—treating smaller stems as intermediate steps during the formation of longer stems. Second, theoretical work [9,10,12,13] capturing polyethylene crystallization phenomenology has often discretized polymer chains into segments ∼3–9 methylene units in length, which is comparable to the size of the coarse-grain beads used in this study. In this vein, single-bead stems are comparable to relevant length scales for probing polyethylene crystallization, and could serve as intermediates during polymer crystallization.

We also performed a substantial detailed analysis of our crystallization simulations to validate the inclusion of the single-bead stems in the current study. For reference, the simulation-based literature on polymer crystallization (e.g., [16,18,19,20,24,25,28,30,34,38,41,42,48]) does not provide a clear precedent as to whether or not such short stems should be included when analyzing polymer crystallization simulations. In particular, some studies include such short stems [18,24,25,28,38,41,42,48], other studies exclude them by invoking a minimum length criterion [16,21,22,34], and still other studies allow for stems of variable length and use a length criterion to assign some stems as crystalline [19,20,30]. Therefore, we assessed the role of single-bead stems in the formation of large, post-critical crystal clusters. Based on earlier work [38], the approximate critical nucleus size (i.e., location of the free-energy barrier to crystallization) for the conditions and molecular model used in this study corresponds to a cluster size of ∼200 coarse-grain beads (i.e., ∼600 carbon atoms); see ref. [38] for more details concerning this estimate. In turn, as part of the cluster analysis for the current study, we traced clusters greater than or equal to the critical nucleus size back to their originating clusters by assessing the overlap, in terms of constituent particle indices, between clusters extracted from the successive snapshots of a given simulation. This analysis revealed that individual single-bead stems can serve as the starting point for the formation of clusters that ultimately become post-critical. These results provide a post-hoc demonstration that single-bead stems are an important intermediate structural motif en route to the formation of larger structures during polymer crystallization. It is thus both appropriate and physically reasonable that the current study include single-bead stems.

For the remainder of this study, nuclei and stem lengths are generally discussed in terms of the number of corresponding carbon atoms in order to facilitate comparisons with previous work. Stem lengths are also reported in terms of their spatial (nm) lengths, which were estimated using the bond length (CM–CM) and bead width (CM) for the SDK model. Throughout this study, various properties are presented as functions of cluster size (e.g., maximum stem length). Each of these profiles was constructed by first extracting the specified property for each nucleus, and then performing conditional averaging over the extracted values according to cluster size.

## 3. Results and Discussion

The crystal cluster shown in Figure 1 corresponds to 600 carbon atoms, the approximate critical nucleus size for the conditions and molecular model used in this study (see [38]). The varying stem lengths in Figure 1 along with similar simulation snapshots from previous studies [17,19,20,27,28,29,30,31,32,33,34] highlight that structural non-uniformity (heterogeneity) is present at the early stages of crystallization in a monodisperse polymer melt. This study contributes a detailed quantitative characterization and discussion of this structural heterogeneity.

**Figure 2:**
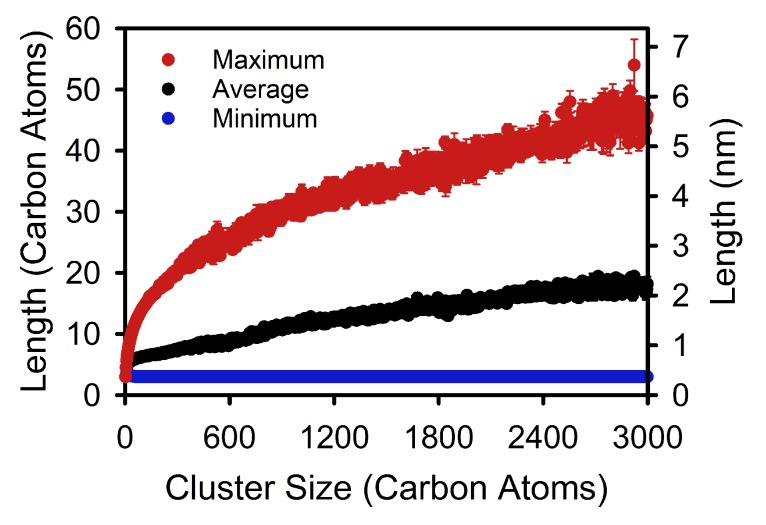
Variation in minimum, maximum, and average stem lengths as a function of crystal cluster size. Each data point corresponds to a size-dependent conditional average for a given nucleus size. For example, the maximum (red) point at 600 carbon atoms indicates the average maximum stem length of clusters corresponding to 600 carbon atoms. The error bars indicate standard errors, which are smaller than the symbol size for the minimum and average data.

Consistent with previous work [14,20], average stem lengths increase continuously with nucleus size across the critical nucleus size (see Figure 2), indicating that nuclei exhibit three dimensional growth and continue to thicken even once they are post-critical. Maximum stem lengths also increase as nucleation proceeds, but initially at a much greater rate than average stem lengths (Figure 2). The continued increase in maximum stem length during post-critical growth suggests that the lamellar thicknesses measured by experimental techniques are attained through post-critical processes. In contrast, minimum stem lengths stay at ∼3 carbon atoms, aligning with previous work indicating that polymer crystals have tapered growth fronts [16,18,19]. Taken together, the results in Figure 2 reveal that the distribution of possible stem lengths broadens rapidly during the pre-critical stages of crystallization, and then more gradually upon transitioning to post-critical growth.

**Figure 3:**
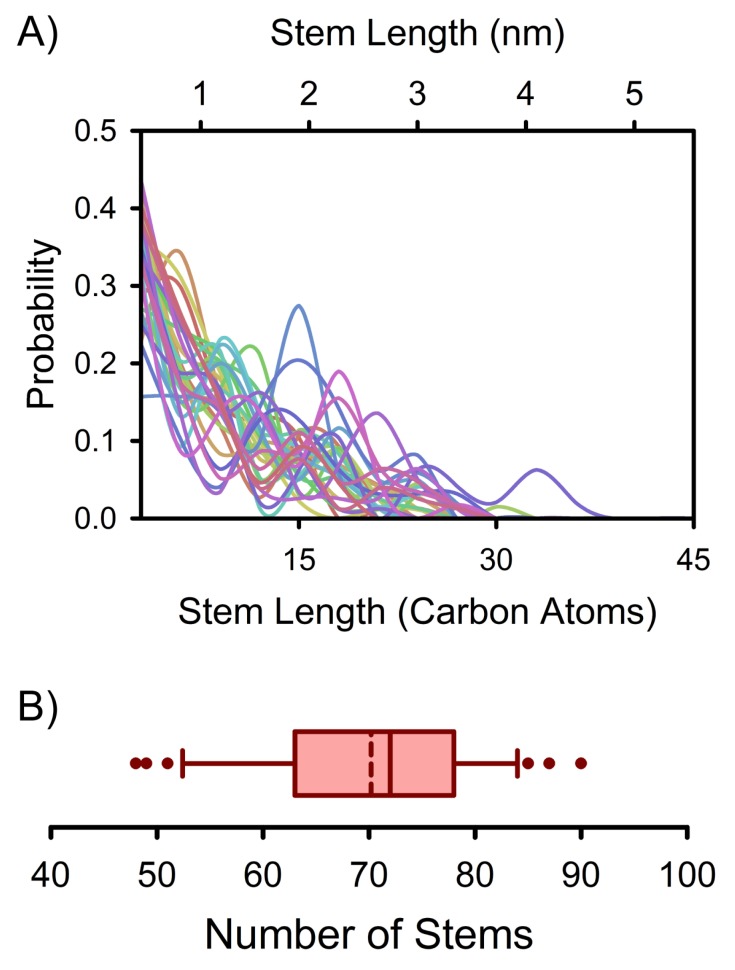
Stem distributions for clusters corresponding to 600 carbon atoms. (**A**) Overlaid stem length probability distributions. Each cluster is indicated with a different color. The stem length axis starts at 3 carbon atoms, the smallest possible stem based on the model and definitions used in this study. Each probability distribution has been represented using a spline curve to help guide the eyes; (**B**) A box-and-whisker plot of the number of stems composing the clusters represented in (**A**). The left and right bounds of the box indicate the 25th and 75th percentiles for the number of constituent stems. The central dashed and solid lines are the average and median numbers of constituent stems. The left and right error bars indicate the 10th and 90th percentiles. The dots indicate outliers not contained in the 10th to 90th percentile region.

In addition to intra-nucleus stem length variations as evidenced by Figure 1 and Figure 2, there are also differences between individual nuclei. In fact, the exact distribution of stem lengths can vary substantially between nuclei even when comparing nuclei that are the same size (see Figure 3A). The distributions in Figure 3A qualitatively differ from those previously reported in crystal growth studies and for mature lamella (e.g., [16,24,25,28]), highlighting the distinction between polymer crystal nucleation and growth. Consistent with the various stem length distributions in Figure 3A, nuclei of a particular size can also differ markedly in terms of their number of constituent stems (Figure 3B). There are thus both intra-nucleus and inter-nucleus structural variations during polymer crystallization in monodisperse melts.

**Figure 4:**
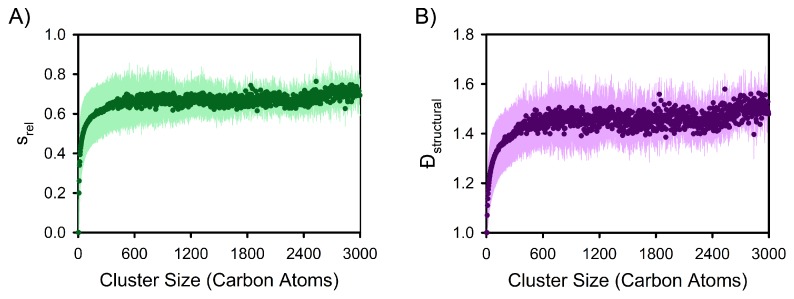
Relative variation in stem length distributions. (**A**) Relative standard deviation in stem lengths (i.e., stem length standard deviation divided by mean stem length) as a function of cluster size. (**B**) Average structural dispersity of nuclei during crystallization; see main text for details and interpretation. In both panels, data points indicate the average values associated with different cluster sizes while the pale-colored areas indicate the corresponding standard deviations.

Given that average stem lengths increase with crystal size (Figure 2), relative metrics were used to quantitatively characterize the evolution of stem length distributions during the nucleation process (i.e., across nuclei of differing size). For example, the average relative standard deviation in the constituent stem lengths of nuclei ($s_{rel}$) and its evolution with cluster size were calculated based on the clusters extracted from the simulations (see Figure 4A). In turn, while very small clusters (i.e., clusters composed of a single stem) exhibit no structural heterogeneity (i.e., $s_{rel}=0$), relative structural heterogeneity increases rapidly with cluster size before stabilizing in the vicinity of the critical nucleus with $s_{rel}>0.6$. Importantly and consistent with Figure 3A, the results in Figure 4A quantitatively capture that each individual nucleus is generally composed of numerous stems of varying length, and thus exhibits substantial structural heterogeneity. Larger clusters are not composed of stems of differing but constant lengths; such a scenario would require $s_{rel}=0$.

We now introduce the concept of the structural dispersity as a physical analog of dispersity (a metric commonly used in synthetic polymer chemistry) in order to place structural heterogeneity in nascent polymer crystals on an equal conceptual footing with chemical heterogeneity arising during polymer synthesis. More specifically, the relative distribution of chain lengths in a polymer sample is commonly captured using dispersity (Đ) as calculated according to:

(1) $$Đ=\frac{{\bar{M}}_{w}}{{\bar{M}}_{n}}$$

with ${\bar{M}}_{w}$ and ${\bar{M}}_{n}$ being the weight-average and number-average molar masses of the constituent polymer chains as calculated according to:

(2) $${\bar{M}}_{w}=\frac{\sum_{i}N_{i}M_{i}^{2}}{\sum_{i}N_{i}M_{i}}$$

(3) $${\bar{M}}_{n}=\frac{\sum_{i}N_{i}M_{i}}{\sum_{i}N_{i}}$$

In Equations (2) and (3), $M_{i}$ is a particular molar mass, $N_{i}$ is the number of polymer chains with a molar mass of $M_{i}$, and the sums are over all polymer chain molar masses in the sample. Monodisperse (uniform) polymer samples, like the polymer melts considered in this study, have Đ = 1. The structural dispersity (Đ_structural_) of a nucleus can be calculated based on Equations (1)–(3) by replacing the polymer chain molar masses of a sample ($M_{i}$) with the lengths of the constituent stems of a nucleus (in terms of the number of constituent monomers) and revising the summations accordingly. In turn, structural dispersity is a quantitative metric for probing the relative distribution of stem lengths in a polymer nucleus or crystal. More specifically, a lamella comprising stems of uniform length exhibits Đ_structural_ = 1, as does a nucleus composed of a single stem. The latter scenario is why the very small clusters extracted from the nucleation simulations exhibit Đ_structural_ in the vicinity of 1 (see Figure 4B). Conversely, large Đ_structural_ values indicate relatively broad stem length distributions.

At this point, it is worth noting that it can be challenging to compare molecular weight distributions using Đ values [52,53,54] as the dispersity of a polymer sample is related to the standard deviation in the molecular masses of the constituent polymer chains (σ) according to Equation (4) [52].

(4) $$Đ={\left(\frac{\sigma }{{\bar{M}}_{n}}\right)}^{2}+1$$

Importantly, the fraction in Equation (4) corresponds to the relative standard deviation in the molecular masses of the polymer chains. As such, it is not appropriate to interpret Đ values as absolute measures of polymer molecular mass distributions. However, the goal of this study is to consider relative changes in stem length distributions, so it is reasonable to consider Đ_structural_ values. Moreover, in analogy to Equation (4), $s_{rel}$ and Đ_structural_ are related according to:

(5) $$Đ=s_{rel}^{2}+1$$

As such, Đ_structural_ values initially increase rapidly with cluster size before leveling off as the critical nucleus size is approached (Figure 4B) in accordance with $s_{rel}$ values (Figure 4A). However, by switching from $s_{rel}$ values to Đ_structural_ values, it is possible to compare the heterogeneity arising during polymer crystallization to that of polymer synthesis. In particular, nuclei in the vicinity of the critical nucleus exhibit Đ_structural_ values in the range of ∼1.4–1.6 (see Figure 4B), which is comparable to the range of Đ values associated with polymerization reactions (e.g., see [55] and references therein).

Minimizing chain dispersity has been the focus of much research on polymerization, and it is possible to achieve synthetic polymers and biopolymers with narrow molar mass distributions (e.g., via living polymerization) [56,57]. Some work [58] has conjectured that monodisperse polymers could enable the attainment of materials with close to 100% crystallinity, something notoriously difficult to achieve experimentally for most polymers. Note that such conjectures are based on short unentangled polymer species crystallizing under mild conditions, whereas industrial polymer specimens are typically composed of long entangled polymer chains and are generally crystallized under high driving-force conditions (e.g., with deep quenches). While most researchers in the polymer community are not seeking to synthesize monodisperse polymers to achieve perfectly crystalline polymeric materials, the aforementioned conjecture is nevertheless emblematic of the prevailing idea that greater control over chain dispersity will yield greater control over the composition and structure of polymeric materials, and thus their properties and performance. However, such thinking neglects the structural dispersity that arises during polymer crystallization. This study quantitatively demonstrates that monodisperse long-chain entangled melts crystallize at high driving forces via the formation of nanoscale nuclei that are structurally polydisperse. Consequently, achieving monodisperse polymer samples is a necessary but not sufficient condition for achieving physical uniformity. In order to fully tailor polymeric materials, strategies must be developed to control both chain (chemical) and structural dispersities.

Both Đ_structural_ and $s_{rel}$ values are related to the second central moment of the stem length distributions of nuclei, and they stabilize in the vicinity of the critical nucleus size (i.e., ∼600 carbon atoms), as can be seen in Figure 4. These results suggest that structural dispersity selection may be connected to polymer nucleation processes. Metrics probing the higher central moments of stem length distributions reinforce that the critical nucleus size corresponds to an approximate cross-over point in terms of the properties of stem length distributions. More specifically, skewness ($\gamma_{1}$) and excess kurtosis ($b_{2}$) depend on the third and fourth central moments of a distribution, and they were calculated for each cluster’s stem length distribution according to Equations (6) and (7), respectively.

(6) $$\gamma_{1}=\frac{\langle {\left(x-\bar{x}\right)}^{3}\rangle }{s^{3}}$$

(7) $$b_{2}=\frac{\langle {\left(x-\bar{x}\right)}^{4}\rangle }{s^{4}}-3$$

**Figure 5:**
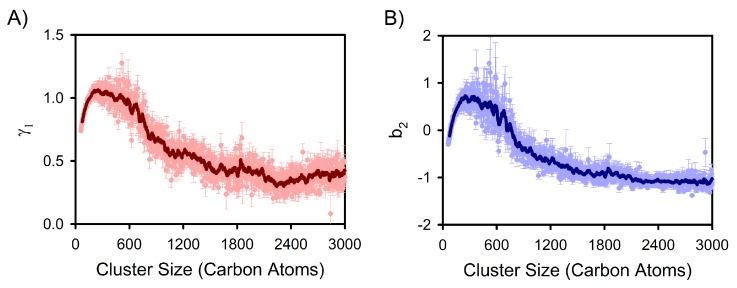
The skewness (**A**) and excess kurtosis (**B**) of stem distributions during polymer crystal nucleation and subsequent growth. In each panel, the dark curve corresponds to a rolling average with a period of 30 carbon atoms to guide the eye. The pale data points correspond to the underlying values for individual nucleus sizes, and the error bars are standard error estimates.

In Equations (6) and (7), the angular brackets indicate averaging over the constituent stems of a cluster, *x* is the length of a particular constituent stem, $\bar{x}$ is the average stem length for the cluster, and *s* is the standard deviation of the stem length distribution for the cluster. Note that the factor of 3 in Equation (7) ensures that a normal distribution has an excess kurtosis of zero. In turn, average skewness and excess kurtosis were determined as functions of nucleus size based on the clusters extracted from this study’s nucleation simulations (see Figure 5A,B). On average, both pre-critical and post-critical nuclei display positive $\gamma_{1}$ values (Figure 5A), indicating long right tails in their stem length distributions, which is consistent with the distributions shown in Figure 3A. Importantly, there is a pre-critical maximum in the skewness of stem length distributions, and an approximate inflection point in the vicinity of the critical nucleus size (∼600 carbon atoms) with $\gamma_{1}$ values continuing to decrease with increasing cluster size. Excess kurtosis values exhibit similar behavior (Figure 5B). For reference, a positive $b_{2}$ value is indicative of a tighter, more peaked distribution (with respect to a normal distribution) whereas negative values are indicative of flatter distributions. The transition from positive to negative $b_{2}$ values in the vicinity of the critical nucleus size arises from the increasing prevalence of longer stems (i.e., development of a flat right tail in stem length distributions). Importantly, both $\gamma_{1}$ and $b_{2}$, along with D_structural_ and $s_{rel}$, all indicate that the pre-critical and post-critical regimes of polymer crystallization display distinct behaviors in terms of stem length distributions. As such, small nuclei are not simply smaller versions of larger-scale crystals, which aligns with insights from previous work monitoring the shape of nascent polymer crystals [38].

Previous work [42] has demonstrated that nucleation in entangled polymer melts at high-driving forces (i.e., for the types of systems considered in this study) is a local event guided by local environments. Nucleation rates for long- and short-chain systems similarly indicate that nucleation is a local event [14]. Moreover, Sommer and colleagues [20,30,59] have previously demonstrated that local entanglement lengths in metastable melts directly influence stem lengths during polymer crystallization. Still other work [23] has connected stem lengths to the degree of disentanglement during polymer crystallization while work [60] on the in vacuo crystallization of isolated polyethylene chains highlights that the distribution of all-trans segment lengths can vary with thermal history. Consequently, it is reasonable to conjecture that structural dispersity is governed by the properties of supercooled polymer melts and solutions (e.g., perhaps local entanglement lengths and environments prior to crystallization), and that factors influencing the characteristics of metastable melts and solutions (e.g., quench sequences, flow, and additives) will likely modulate stem length distributions.

## 4. Conclusions

The chemistry community has focused extensively on controlling chain length distributions (i.e., chemical dispersity) during polymer synthesis. However, the continued pursuit of lower chemical dispersities will likely not yield the desired control of polymeric materials unless the chemistry community also develops strategies to guide polymer crystallization down to its earliest stages. Our results demonstrate that controlling crystallization cannot simply take place at macroscopic or mesoscopic scales; crystallization must be controlled down to the nanoscale processes through which it is initiated. Even in monodisperse polymer melts, individual nuclei exhibit substantial nanoscale stem length variations, while nuclei of a given size can have markedly different stem length distributions. Structural heterogeneity and structural dispersity are thus important characteristics of polymer crystal nucleation. Consequently, fully tailoring polymeric materials requires strategies for minimizing intra-nucleus and inter-nucleus structural variations on sub-nanometer and nanometer levels.

This study has also revealed that various quantitative properties of stem length distributions (including relative standard deviation, skewness, excess kurtosis, and structural dispersity) exhibit distinct pre-critical and post-critical behaviors with cross-overs in the vicinity of the critical nucleus size. As such, our quantitative characterizations of stem length distributions and their average evolution reinforce that nucleation is phenomenologically distinct from polymer crystal growth, suggesting that insights from crystal growth studies are not necessarily relevant to attempts to control polymer crystal nucleation. By introducing structural dispersity and numerically analyzing stem length distributions more generally, this study provides a fresh quantitative lens for comparing and understanding both polymer crystal nucleation and growth moving forward. Interestingly, the structural dispersity values obtained in this study for clusters in the vicinity of the critical nucleus size are comparable to dispersity values arising from polymerization reactions. As illustrated by a recent perspective article [61], it remains an outstanding challenge to establish metrics that capture processing-induced polymer heterogeneities and behaviors as well as quantitative connections between polymer conformations and processing. Structural dispersities and stem length distributions may provide a quantitative basis for tackling this challenge as computational studies increasingly probe the molecular-level details of polymer processing phenomena and their effects on crystallization.

## Figures and Tables

**Table 1:** Simulation details for the nucleation simulations. The unscaled times indicate the times that were used in the LAMMPS input files, whereas the scaled times include the scaling factor of 4.07.

**Simulation Software**	LAMMPS [39]
**Coarse-Grain Model**	SDK model [40] (CM and CT beads)
**Simulation Ensemble**	NPT (isobaric-isothermal)
**Temperature**	285 K
**Thermostat Details**	Nosé–Hoover [44,45] chain thermostats [46] (4-member chains, coupling constants: 1 ps unscaled time)
**Pressure**	1 atm
**Barostat Details**	Anisotropic chain barostats [43] (4-member chains, coupling constants: 10 ps unscaled time)
**Boundary Conditions**	Periodic
**Lennard-Jones Cutoff**	1.5 nm
**Simulation Length**	200,000,000 time steps
**Time Step Length**	5 fs unscaled time (20.35 fs scaled time)
